# A Pectin Methylesterase *ZmPme3* Is Expressed in *Gametophyte factor1-s (Ga1-s)* Silks and Maps to that Locus in Maize (*Zea mays* L.)

**DOI:** 10.3389/fpls.2017.01926

**Published:** 2017-11-07

**Authors:** Adrienne N. Moran Lauter, Michael G. Muszynski, Ryan D. Huffman, M. Paul Scott

**Affiliations:** ^1^Corn Insects and Crop Genetics Research Unit, USDA-ARS, Ames, IA, United States; ^2^Department of Tropical Plant and Soil Sciences, University of Hawaii at Manoa, Honolulu, HI, United States; ^3^Molecular, Cellular and Developmental Biology Program, Iowa State University, Ames, IA, United States

**Keywords:** gametophytic incompatibility, pollen, pistil, *Zea mays*, pectin methylesterase

## Abstract

The *ga1* locus of maize confers unilateral cross incompatibility, preventing cross pollination between females carrying the incompatible *Ga1-s* allele and males not carrying a corresponding compatible allele. To characterize this system at the molecular level, we carried out a transcript profiling experiment in which silks from near isogenic lines carrying the *Ga1-s* and *ga1* alleles were compared. While several differentially expressed genes were identified, only one mapped to the known location of *ga1*. This gene is a pectin methylesterase (PME), which we designated as *ZmPme3*, and is present and expressed only in *Ga1*-*s* genotypes. While a functional ZmPME3 is not present in the *ga1* genotypes examined, a pectin methylesterase gene cluster is found in *ga1* genotypes. The gene cluster in W22 contains 58 tandem full-length or partial PME pseudo genes. These data combined with a wealth of previously published data on the involvement of PMEs in pollen tube growth suggest a role for cell wall modification enzymes in the pollen exclusion component of *Ga1-s* gametophytic incompatibility. Consistent with this role, a third allele which lacks the female function of *Ga1-s, Ga1-m*, has a mutationally inactivated version of *ZmPme3*.

## Introduction

Maize is a wind-pollinated plant, where pollen grains released from anthers on the tassel are dispersed to the female silks on the ear. Unlike many other plants, maize male and female flowers are separate, resulting in high rates of cross-pollination. Several cross incompatibility systems have been identified in maize. These systems may have provided a reproductive barrier between maize and teosinte (Evans and Kermicle, 2001; Kermicle and Evans, 2005) in the evolution of modern maize. Cross incompatibility systems also have practical application in limiting undesired pollen transmission between different market classes of corn, such as dent and popcorn (Nelson, 1953).

One of the first genetic studies in maize following the rediscovery of Mendel's laws was a study of cross incompatibility between maize and popcorn (Correns, 1901). The genetics of this and other cross incompatibility systems in maize have been studied intensely since then and have been reviewed (Nelson, 1994). One of these systems was designated Gametophyte factor (Mangelsdorf and Jones, 1926) or *Ga* (now called *Ga1*) because of the involvement of the gamete in cross incompatibility. In the *Ga1* system, cross incompatibility is conferred by a factor in silks of plants carrying the Ga1-strong (*Ga1-s*) allele, unless the male gamete also carries the *Ga1-s* allele. Cross incompatibility is therefore unilateral, providing a reproductive barrier between *ga1* pollen and silks containing *Ga1-s*, but not between *Ga1-s* pollen and *ga1*/*ga1* silks. The *Ga1-s* allele is common in popcorn and annual teosinte (Kermicle et al., 2006), while most other maize varieties carry the *ga1* allele and are therefore susceptible to the fertilization barrier imposed by *Ga1-s*. While most dent corn can freely cross pollinate, crosses between dent corn and popcorn must usually be carried out with the popcorn as the male parent.

The *Ga1-s* allele performs two functions, 1. Excluding pollen not carrying the *Ga1-s* allele and 2. Pollinating *Ga1-s* silks. We refer to these functions as the female and male functions, respectively. Cross compatibility is thus conferred by the lack of an incompatibility system in both parents as in the case of *ga1* or by a compatible interaction between the male and female factors of the *Ga1-s* allele (Jones, 1924). Characterization of the *Ga1-m* allele containing only the male function (Jimenez and Nelson, 1965) validated the idea that the *ga1* locus conferred two functions and further demonstrated that these functions are genetically separable. *Ga1-m/Ga1-m* plants can overcome the *Ga1-s* crossing barrier, but their silks are receptive to pollination by all *ga1* alleles; *Ga1-s, Ga1-m* and *ga1*. Thus, *Ga1-m* performs the male function but not the female function of the locus.

Kermicle and Evans (2005) used disomic pollen grains derived from a tertiary trisomic plant carrying both *Ga1-s* and *ga1* to demonstrate that presence of *Ga1-s* in pollen is sufficient to overcome the pollination barrier. This is consistent with a model requiring congruity of the pollen and silk alleles, as opposed to the alternative hypothesis of active rejection of *ga1* by the *Ga1-s* allele in silk tissue. One possibility raised by this observation is that the *ga1* allele is actually a null allele. If *ga1* is null due to the absence of an active gene that is present only in *Ga1-s* genotypes, identification of the gene of interest will be difficult because all currently sequenced and assembled genomes carry only the *ga1* allele.

Maize pollen tubes have been shown to have an altered growth rate (Zhang et al., 2012) and morphology when grown on incompatible silks (Lu et al., 2014). In the *Ga1* system, this morphology consists of an erratic growth pattern, with the pollen tube often outside of the transmitting tract of the pistil. Data suggest this behavior may be a result of altered pollen tube cell wall growth (Bosch and Hepler, 2006). Several studies illustrate the importance of pectin methylesterase and pectin methylesterase inhibitors in regulating pollen tube growth by altering the balance between strength and plasticity of the apical cell wall of the pollen tube (reviewed in Krichevsky et al., 2007; Zonia and Munnik, 2009).

The *Ga1-s* female function has been genetically mapped (Bloom and Holland, 2011), as has the male function (Zhang et al., 2012). In spite of mapping different functions, the region identified in these studies overlaps substantially. The most precise mapping data suggests that the male function is contained on a 100 kb region of maize chromosome 4 based on B73 RefGen_v2 (Liu et al., 2014). Despite the presence of only a few candidate genes in that region, the causative *Ga1-s* gene has yet to be identified. However if *Ga1-s* is not present in *ga1* backgrounds, identification through a candidate gene approach based on *ga1* genomes such as B73 may not be effective.

The objective of this study is to gain insight into the molecular mechanism of *Ga1* function. We conducted a transcriptomic study of unpollinated silks from *Ga1-s* (W22) and *ga1* (W22) which identified *ZmPme3* as a candidate gene for the female function of *Ga1-s*. Proteomics, genetic mapping and genomic sequences of *ga1* alleles support this hypothesis. The results of this study, together with data in the literature suggest a role for cell wall enzymes in the molecular genetic function of the *Ga1* system. This discovery allows for new avenues for testing hypotheses about the molecular mechanism of gametophytic incompatibility systems.

## Materials and methods

### Aniline blue staining of pollen tubes

**Figure 1:**
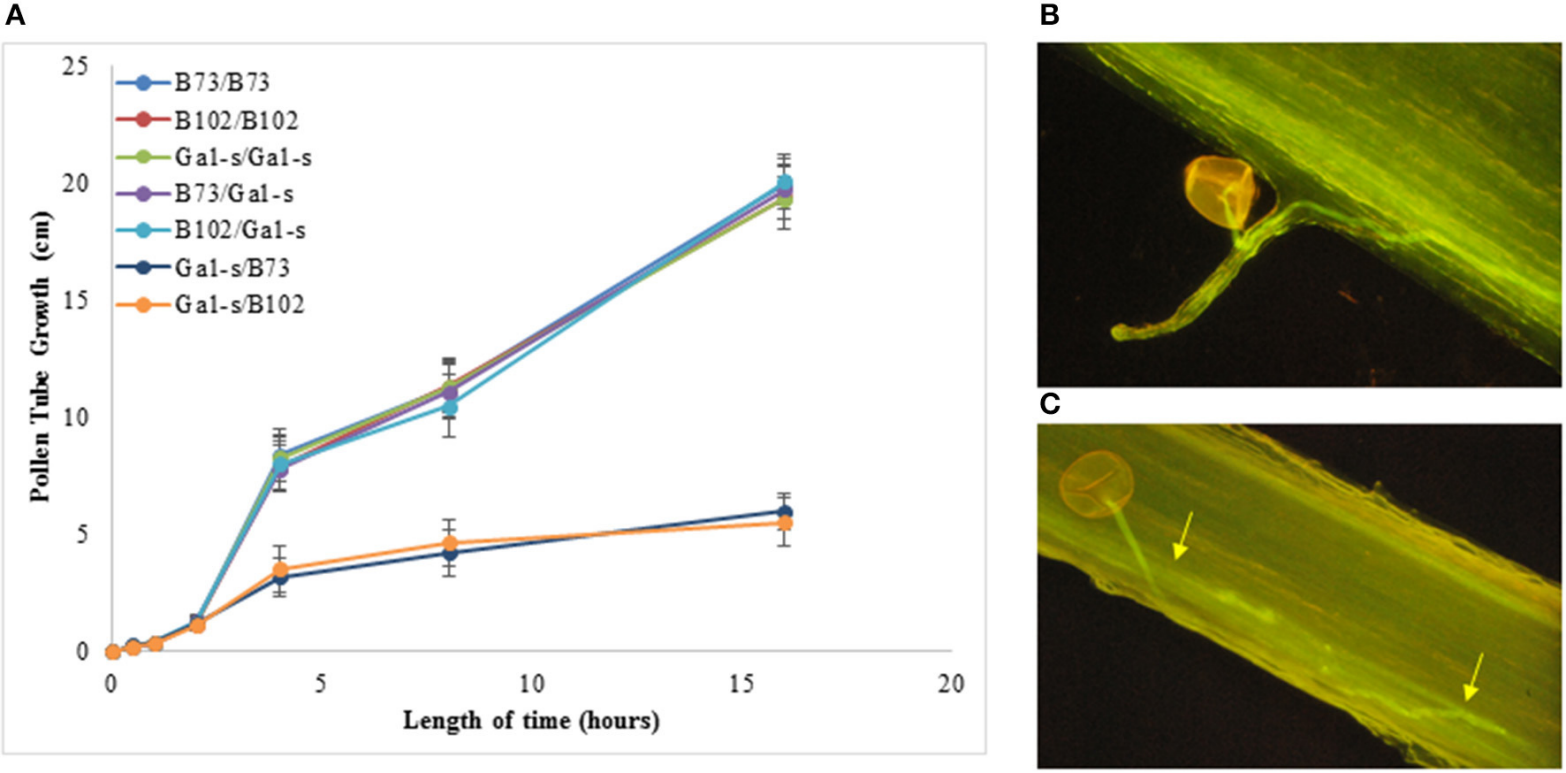
Pollen tube growth in compatible vs. incompatible pollinations. Pollen tubes from compatible and incompatible pollinations were fixed and stained with aniline blue at fixed times after pollination **(A)** Length of pollen tubes over time in compatible vs. incompatible pollinations. Error bars represent the standard deviation for three independent experiments. **(B)** Aniline blue staining *in vivo* of pollen tubes in a compatible pollination. Pollen tubes grow straight once inside the transmitting tract (40X magnification). **(C)** Aniline blue staining *in vivo* of pollen tubes in an incompatible pollination. Incompatible pollen tubes do not grow straight and are sometimes seen exiting and reentering the transmitting tract (arrows).

Pollen tubes shown in Figure 1 were visualized following silk fixation using a combination of methods performed by Lausser et al. (2010) and Zhang et al. (2012). Briefly, pollen tubes were allowed to germinate for a predetermined time before silk tissue was harvested and incubated in FAA (10:85:5 v/v/v formaldehyde: 95% ethanol: acetic acid) for 24 h at 4°C. Silks were then slowly rehydrated by washing in 75, 50, 25% ethanol for 3 min each. Samples were washed in 0.1 M potassium phosphate buffer (pH 8.0) before a 2 h incubation in 8 M NaOH. Silk tissue was stained in 0.1% aniline blue (Fisher Scientific, Pittsburgh, PA) dissolved in 0.1 potassium phosphate overnight at 4°C. Silks were placed on glass slides and pollen tubes were visualized at near UV excitation using a fluorescent microscope (Nikon Diaphot 300). At least 10 silks per plant were measured and three biological replicates were completed for each experiment. In transmitting tracts with more than one pollen tube germinated, measurements were recorded from the pollen grain closest to the ovule.

### RNA isolation for RNA-seq and qRT-PCR

W22 (*ga1*) and *Ga1-s* (W22) (Maize Genetic Stock Center number 401D) plants were grown in the greenhouse at Iowa State University. Three biological replicates of unpollinated, emerged silks from each genotype were collected for RNA isolation. Following isolation, RNA was sent to the DNA facility at Iowa State University for single-end RNA sequencing on Illumina MiSeq with a read length of 50 bp.

For qRT-PCR analysis, W22 (*ga1*), *Ga1-s* (W22) and Hp301 were grown in the USDA greenhouse at Iowa State University. Five to seven biological replicates of unpollinated, emerged silks from each genotype were collected for RNA isolation by Qiagen RNeasy Plant kit.

### RNA-seq and bioinformatic analysis

Sequencing was performed at the Iowa State University on a MiSeq. Libraries were sequenced for 51 cycles. TopHat (version 2.0.3, Trapnell et al., 2009) was used to align reads to the B73 reference genome (version 3 ref). The program samtools (Li et al., 2009) was used to remove unreliably mapped reads. The resulting mapping files (bam) were imported into the statistical program R (R Core Team, 2016) using the Bioconductor package Rsamtools (Morgan et al., 2010). Mapped reads were submitted to NCBI SRA under BioProject PRJNA382364.

The R graphics program ggplot2 (Wickham, 2009) was used to compare sample replicates for technical reproducibility (data not shown). Samples of low quality were not used in further analyses. The Bioconductor package edgeR (Robinson and Smyth, 2007, 2008; Robinson et al., 2010; McCarthy et al., 2012) was used for single factor, pairwise comparisons to calculate normalization factors, estimate tagwise dispersion and determine differential expression (DEG) based in comparison to the reference genome B73_v3. 1452 DEG were identified at a cutoff of *p* > 0.05. (Table S3).

RNA-Seq reads were also assembled *de novo* through the Trinity package. RSEM was used to estimate abundance after mapping reads back to assembled transcripts using bowtie2. The raw counts for transcripts were then normalized (upper quartile) and differential gene expression (DEG) analysis was performed (three independent methods were used: edgeR, voom, DESeq2). EdgeR results are presented in this paper. ORFs were predicted from these transcripts and predicted peptides were generated. Annotation of the assembled transcripts was performed using Trinnotate which uses a curated dataset from swissprot to predict function as well as the pfam database, tmhmm, signalp, and gene ontology for additional annotation. Some sequences that were reported as differentially expressed most likely resulted from a sequence polymorphism between the two genotypes sequenced because a corresponding transcript with minor sequence differences and approximately the same read count was also found on this list. These transcripts were manually removed from the gene list shown in Table 1, but are still present in Table S1.

**Table 1:** Most significant DEG of *de novo* assembled transcripts.

**GeneID**	**Log2 Fold Change**	**FDR**	**Merged annotation**	**Chromosome**
TRINITY_DN41247_c0_g1	−9.004	4.00E-121	Putative pectinesterase/pectinesterase inhibitor 38;	4^*^
TRINITY_DN17388_c1_g1	−9.868	5.64E-114	NA (abscisic acid protein homolog aba1)	10
TRINITY_DN35119_c0_g2	−13.410	9.93E-81	Retrovirus-related Pol polyprotein from transposon TNT 1-94;	
TRINITY_DN16496_c0_g1	8.704	6.91E-76	Cysteine-rich receptor-like protein kinase 10;	6
TRINITY_DN13019_c0_g1	−10.384	4.72E-74	NA	3
TRINITY_DN49285_c0_g1	−7.057	2.16E-65	Alpha-humulene synthase;	1
TRINITY_DN11568_c1_g1	7.665	4.09E-56	Probable polygalacturonase;	8
TRINITY_DN10723_c0_g1	−10.277	1.37E-54	NA	3
TRINITY_DN9448_c0_g1	7.460	4.22E-53	Autonomous transposable element EN-1 mosaic protein;	6
TRINITY_DN29956_c0_g1	−8.817	1.05E-50	Transposon Tf2-9 polyprotein;	
TRINITY_DN35208_c1_g1	6.888	1.21E-49	NA	6
TRINITY_DN9608_c0_g1	−8.402	1.53E-47	NA	2
TRINITY_DN47379_c0_g1	7.777	2.59E-47	3beta-hydroxysteroid-dehydrogenase/decarboxylase isoform 1;	1
TRINITY_DN35119_c0_g1	−8.863	3.05E-47	Retrovirus-related Pol polyprotein from transposon TNT 1-94;	
TRINITY_DN8884_c0_g1	−7.909	8.50E-47	NA	
TRINITY_DN48292_c0_g1	−8.506	8.35E-45	NA	
TRINITY_DN23825_c0_g1	−11.575	1.88E-44	Transposon Tf2-9 polyprotein;	
TRINITY_DN35959_c0_g1	8.383	1.54E-43	MuDR family transposase	10
TRINITY_DN14428_c1_g1	−11.621	2.10E-43	Serine/threonine protein phosphatase 2A 55 kDa regulatory subunit B alpha isoform;	9
TRINITY_DN17937_c0_g1	−6.926	5.04E-43	NA	
TRINITY_DN16813_c0_g1	6.165	1.06E-42	Peroxidase 15 {ECO:0000303|PubMed:17936696};	5
TRINITY_DN2380_c0_g1	−9.674	1.31E-42	Cinnamoyl-CoA reductase 2;	8
TRINITY_DN14093_c0_g1	5.960	1.84E-42	NA	8
TRINITY_DN25668_c0_g1	−12.040	8.53E-42	Hevein-like preproprotein;	4
TRINITY_DN8736_c0_g1	−7.303	1.62E-40	Receptor-like serine/threonine-protein kinase At3g01300;	6
TRINITY_DN22910_c0_g1	6.321	7.13E-40	Aspartic proteinase nepenthesin-2;	10
TRINITY_DN19603_c0_g1	6.291	7.41E-40	NA	6
TRINITY_DN24156_c0_g1	−9.386	1.51E-39	NA	2
TRINITY_DN44291_c0_g1	−8.714	1.74E-39	NA	1
TRINITY_DN14200_c0_g1	−4.876	5.86E-39	Replication protein A 70 kDa DNA-binding subunit B;	3
TRINITY_DN43062_c0_g1	−8.025	1.12E-37	Glutathione S-transferase T3;	9
TRINITY_DN41362_c0_g1	−7.207	1.47E-37	NA	1
TRINITY_DN29392_c0_g1	6.357	1.86E-37	NA	1
TRINITY_DN37520_c0_g1	−9.216	4.62E-37	NA	4
TRINITY_DN48873_c0_g1	−9.200	7.21E-37	Plant transposase (Ptta/En/Spm family)	6
TRINITY_DN2409_c0_g2	11.574	8.54E-37	NA	8
TRINITY_DN4369_c0_g1	−10.865	8.10E-36	Probable 4-coumarate–CoA ligase 3;	5
TRINITY_DN2195_c0_g1	5.321	1.11E-35	NA	7
TRINITY_DN10002_c0_g2	5.010	1.56E-35	NA	9
TRINITY_DN5878_c0_g1	11.312	1.75E-35	NA	6

*Top 40 most significant genes. Chromosome location as determined by top BLAST hit to B73 RefGenv3. The asterisks in the Chromosome column indicate BLAST hits were found in the genetically mapped location of Ga1. Where chromosome location is missing, significant BLAST hits were not found in the maize genome. In some cases BLAST hits were found in other organisms that allowed identification of the gene*.

### qRT-PCR of *ZmPme3*

Equal amounts (0.5 ng) of total RNA were used in a one step RT/qPCR reaction with SuperScript® III Platinum® SYBR® Green One-Step qRT-PCR kit (Invitrogen #11736-059). 18S was used as an internal control and *ZmPme3* primers (Table S4) were used to detect transcripts. *ZmPme3* ΔCt levels were normalized to 18S RNA levels and *ZmPme3* transcript levels in *Ga1-s* and Hp301 are expressed as relative to those of W22.

### Amplification and sequencing of *ZmPme3* in *Ga1-s* lines

PCR primers were designed using the *de novo* assembled transcript sequence to amplify the corresponding genomic sequence in four pieces (Table S4). Using genomic DNA from the maize genetic stock center stock 401D which carries the genotype *Ga1-s/Ga1-s* in a W22 background, Hp301 which is a popcorn line known to be *Ga1-s/Ga1-s*(obtained from the North Central Region Plant Introduction Station), W22 (*ga1/ga1*)(obtained from the North Central Region Plant Introduction Station) and NC390/NC394(*Ga1-m/Ga1-m*)(provided by Dr. M. Goodman, NCSU), products were amplified and sequenced in both directions at the Iowa State University DNA Facility. NAM B73 × Hp301 seeds were obtained from the Maize Genetics Stock Center (maizecoop.cropsci.uiuc.edu/).

### Genetic mapping of *ZmPme3* in the Hp301xB73 NAM population

The NAM lines used to map the *ga1* locus with recombination breakpoints in the region of *Ga1-s* (Bloom and Holland, 2011) were screened for the presence of the active PME gene sequence by PCR (Table 2). NAM B73 × Hp301 seeds were obtained from the Maize Genetics Stock Center (maizecoop.cropsci.uiuc.edu/). Two primer pairs (PME marker and PME_A, Table S4) which distinguish the active *ZmPme3* from the inactive genes in W22, were used. The PCR results were compared to the published pollen exclusion phenotypes of these lines (Bloom and Holland, 2011).

**Table 2:** Mapping *ZmPme3* to chromosome 4 using the NAM Hp301 × B73 lines that were used to determine the *ga1* locus (Bloom and Holland, 2011).

**NAM line**	**Bloom and Holland phenotype**	***ZmPme3* marker**
z010E0043	Ga1-s	Ga1-s
z010E0046	Ga1-s	Ga1-s
z010E0030	Ga1-s	Ga1-s
z010E0187	Ga1-s	Ga1-s
z010E0003	Ga1-s	Ga1-s
z010E0038	Ga1-s	Ga1-s
z010E0163	Ga1-s	Ga1-s
z010E0065	Ga1-s	Ga1-s
z010E0092	Ga1-s	Ga1-s
z010E0082	Ga1-s	Ga1-s
z010E0064	Ga1-s	Ga1-s
z010E0018	Ga1-s	Ga1-s
z010E0070	Ga1-s	Ga1-s
z010E0178	Ga1-s	Ga1-s
z010E0180	Ga1-s	Ga1-s
z010E0052	Ga1-s	Ga1-s
z010E0050	Ga1-s	Ga1-s
z010E0133	Ga1-s	Ga1-s
z010E0017	Ga1-s	Ga1-s
z010E0189	Ga1-s	Ga1-s
z010E0042	Ga1-s	Ga1-s
z010E0051	Ga1-s	Ga1-s
z010E0047	Inconsistent	Ga1-s
z010E0106	Inconsistent	Ga1-s
z010E0036	ga1	ga1
z010E0061	ga1	ga1
z010E0009	ga1	ga1
z010E0153	ga1	ga1
z010E0012	ga1	ga1
z010E0020	ga1	ga1

*Green indicates a Ga1-s phenotype or determination by ZmPme3 markers and red denotes ga1 lines*.

### Dot plot and alignments of PME pseudo gene cluster members from W22

To visualize the structure of the W22 PME repeat region, the 3 Mb portion of the Chromosome 4 genomic DNA sequence containing regions of PME homology was analyzed using YASS (Noé and Kucherov, 2005) by plotting the sequence against itself (**Figure 3**). Genomic sequences of each PME pseudo gene were extracted from the genomic sequence using the genome coordinates produced by YASS. The resulting PME pseudogene sequences were aligned using BioEdit and Clustal W. The genome coordinates of the aligned sequences was used to produce **Figure 4**.

### Proteomic analysis of *Ga1-s* tissues

Pollen protein extract was produced from 20 mg of mature pollen grains by mortar and pestle (Zhu et al., 2011). Pistil (pre- and post-pollination) protein extract was produced by grinding tissue in liquid nitrogen using a mortar and pestle into a fine powder that was extracted with trichloroacetic acid (Méchin et al., 2007). Pollen and pistil protein extracts were analyzed by the Protein Facility at Iowa State University as a fee-for-service. The isotopic label-free relative quantification method used was as follows; 100 μg of each protein extract was digested in solution using a trypsin/Lys-C protease mix. Samples were dried down and spiked with 250 fmol of peptide retention time calibration (PRTC) standards during reconstitution. PRTC standards contained an equimolar mixture of 15 known peptides to allow for the quantification of unknown peptides. Newly digested peptides were then separated by liquid chromatography before analysis by MS/MS using a Q Exactive Hybrid Quadrupole-Orbitrap Mass Spectrometer (Fisher Scientific, Waltham, MA). The resulting spectral data was compared to theoretical fragmentation patterns using the MASCOT search engine (Matrix Science, London, UK) to identify the most likely protein accessions by considering the highest-scoring peptide sequence for each input fragment and alignment of 2 or more high-scoring peptide sequences to a single accession. A trinity-predicted peptide database from silk transcript *de novo* assembly was used to make protein identifications.

## Results

### Pollen tubes show aberrant growth patterns in incompatible pollinations of maize silks

To confirm pollen tube growth arrest (Lu et al., 2014) and slow growth rate (Zhang et al., 2012) characteristic of *ga1* pollen on *Ga1-s* silks (Lu et al., 2014) in our genetic materials, pollen tubes from compatible and incompatible pollinations were visualized. In compatible pollinations, pollen tubes were straight and grew within the transmitting tract of the silk to the ovule. In incompatible pollinations of *ga1* (W22) pollen on *Ga1-s* (W22) silks, the pollen tubes had a slower rate of growth (Zhang et al., 2012), were not straight and often left the transmitting tract while growing (Figure 1C).

### A pectin methylesterase is expressed in *Ga1-s* and not in *ga1* and is homologous to sequences at the *ga1* locus

To identify candidate genes for the *Ga1-s* allele, we sequenced transcripts from the silks of two near isogenic lines differing at the *ga1* locus (*Ga1-s/Ga1-s* vs *ga1/ga1*) in the inbred background W22. The sequencing reads generated by this study were deposited in the National Center for Biotechnology Short Read Archive (NCBI SRA BioProject accession PRJNA382364). There were 65,780,803 reads from the *ga1/ga1* sample and 65,261,178 reads from the *Ga1-s/Ga1-s* sample. Since genetic experiments suggest that *ga1* may be a null allele, it could be missing from *ga1* genotypes such as W22 and B73 and mapping reads to these genotypes would not be expected to identify transcripts from the *Ga1-s* gene. We therefore processed the transcript data by *de novo* assembly of the pooled RNA reads. There were 69,160 Trinity assembled transcripts and 61,891 “genes” identified. We then identified differentially expressed genes (DEGs) using edgeR with a FDR of < 0.001. At that cutoff, there are 1958 significant DEG, with 1005 of them upregulated in *Ga1-s* silks (Table 1, Table S1). We used Trinnotate and BLAST to identify the assembled transcripts where 706 transcripts had significant BLAST hits, leaving 1252 without identification.

Keeping with the hypothesis that the *Ga1-s* transcripts are missing from *ga1* genotypes, we focused on genes that were upregulated in *Ga1*-*s*. The most significantly differentially expressed (based on FDR) of the genes upregulated in *Ga1-s* is a putative pectinesterase/pectinesterase inhibitor 38. Among the *de novo* assembled transcripts, there is only one *Pme38*-like transcript and the reads used to assemble it came overwhelmingly from *Ga1-s* samples (Supplemental File). A BLAST search was performed against the B73 genome (RefGen3) with the 40 most significant DEG that were up regulated in *Ga1-s* to determine potential genome localizations. Only the pectinesterase gene hit within the *ga1* locus on chromosome 4. A hevein-like preproprotein resides just outside the locus.

Two genes identified in the B73 genome that lie within the 100 kb region of interest on chromosome 4 such as GRMZM2G027021 (GTP-binding protein hflX) and GRMZM2G039983 (Wave-dampened 2) were detected in the *de novo* assembly (Trinity_13706 and Trinity_17477). Both genes appear to be expressed in silks but not differentially. There do not appear to be genotype differences in transcript sequence for Trinity_17477 (Wave-dampened 2) despite SNPs for GRMZM2G039983 genomic sequence between *Ga1-s, Ga1-m* and *ga1* (data not shown).

### *Ga1-s* genotypes have a PME gene that is lacking in *ga1* genotypes

**Figure 2:**
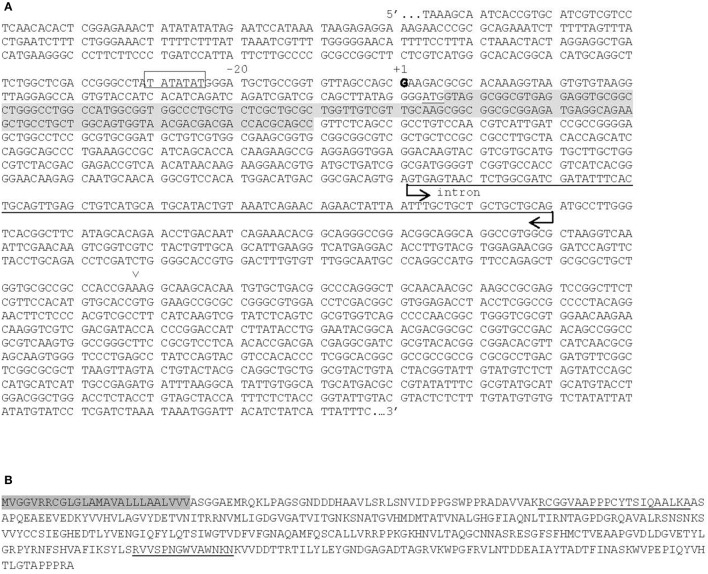
Genomic and predicted protein sequences of *ZmPme3*. **(A)** Genomic sequence of *ZmPme3*, with predicted TATA sequence in a box, +1 base is in bold, ATG start and TGA stop codons are underlined, predicted signal sequence in gray, and intron indicated by arrows. The position of the “GA” insertion in the *Gal-m* hybrid NC390/NC394 is indicated by arrows. **(B)** Predicted protein sequence of ZmPme3 with peptides detected my LC-MS underlined and predicted signal peptide highlighted.

In order to confirm that the *de novo* assembled PME transcript is encoded by a gene present in *Ga1-s* plants and is not a bioinformatic artifact, we used PCR to amplify genomic DNA from *Ga1-s*(W22) and Hp301 (a popcorn line known to be *Ga1-s/Ga1-s*). We amplified a genomic DNA sequence consistent with the computationally derived transcript (Figure 2A). The same PCR reactions were done in *ga1*(W22) and while amplification products were produced, they were not identical in sequence to *Ga1-s* and Hp301 and did not encode functional PMEs. Thus, the hits from the BLAST search with the PME transcript against the B73 genome were likely to be sequences with homology to the PME within the chromosome 4 region of interest. The PME coding sequence from *Ga1-s*(W22) and Hp301 is 1578 bases long with a 99-bp intron and is predicted to encode transcripts identical to the sequence from the *de novo* assembly. We designated the gene encoding this transcript *ZmPme3*. According to the literature, the coding sequence of PME genes range from 1.2 to 3.0 kb long (Wang et al., 2013). We used Hmmer to search for Pfam families within the predicted protein sequence and found only the PME domain (PF01095) with a signal peptide.

We further confirmed the expression data by qRT-PCR. RNA was isolated from unpollinated, exposed silks from W22, *Ga1-s*(W22) and Hp301. Using two primer pairs for *ZmPme3*, relative expression levels were 17- to 97-fold higher in *Ga1-s* genotypes than W22 (Figure S1). ΔCt values for *ZmPme3* from W22 samples were near no template control levels as would be expected if this genotype lacks the *ZmPMme3* gene (Figure S1).

### The gene encoding ZmPme3 maps to the *ga1* locus in an Hp301 × B73 mapping population

While we identified sequences homologous to *ZmPme3* in the genomic region known to contain *ga1* using BLAST, the genome position of *ZmPme3* was not yet clear. To determine whether *ZmPme3* maps to the *ga1* locus, we mapped *ZmPme3* in the Hp301 × B73 RIL population developed for the NAM project (McMullen et al., 2009). Based on previously published information about this population (Bloom and Holland, 2011) we screened the lines used to map the *ga1* locus with recombination breakpoints in the region of *Ga1-s* for the presence of the active PME gene sequence by PCR. *ZmPme3* was detected in the NAM lines that excluded pollen and was not detected in lines that did not exclude pollen (Bloom and Holland, 2011; Table 2). These data confirmed that *ZmPme3* from *Ga1-s* is in the *ga1* interval that Bloom and Holland identified.

### A protein encoded by the *ZmPme3* gene is detectable in *Ga1-s* silks, but not in *ga1* silks

To determine if the ZmPME3 protein showed an expression pattern similar to the *ZmPme3* transcript, protein extracts from mature pollen and silk tissue pre- and post-pollination were subjected to mass spectrometry analysis. Six samples were analyzed, all in the W22 background; *Ga1-s* and *ga1* mature pollen, *Ga1-s* and *ga1* silks, *ga1* silks pollinated with *Ga1-s* pollen (compatible) and *Ga1-s* silks pollinated with *ga1* pollen (incompatible). Identification of the peptides generated was performed by matching resulting peptide masses to the predicted proteins from the *de novo* assembled transcripts of *ZmPme3*. Peptides from ZmPME3 (Trinity_DN41247) were detected in both *Ga1-s* unpollinated and pollinated silks at a coverage of 7.8% (Figure 2B). Peptides from ZmPME3 were detected in neither of the pollen samples nor the *ga1* unpollinated and pollinated silks. We conclude that the ZmPME3 protein expression is consistent with the expression of the *ZmPme3* transcript.

### Plants containing the *Ga1-m* allele contain a non-functional *ZmPme3* gene

The *Ga1-m* allele lacks the female function of the *ga1* locus, so the sequence of *ZmPme3* in this genotype was of great interest. If *ZmPme3* is responsible for the female function of *Ga1-s*, we would expect it to be non-functional in *Ga1-m* genotypes. Through PCR and sequencing of *ZmPme3* in the *Ga1*-*m/Ga1-m* containing hybrid NC390/NC394, we found a 2 base insertion that causes a frameshift resulting in a truncated and likely inactive protein (Figure 2A). This observation is consistent with the hypothesis that *ZmPme3* is involved in the female function of *Ga1-s*.

### Structure of the PME repeat region of chromosome 4 (W22/B73)

The transcript profiling with *de novo* assembly and proteomics experiments presented here show that the *Ga1-s* genotypes examined have a single active differentially expressed PME gene, however we observed BLAST hits to PME sequences in the *ga1* genotypes as well. Our initial observation of PME-like sequences in a *ga1* genotype was when RNA reads were mapped to B73_RefGen v3 (Schnable et al., 2009). The top 40 most significant (lowest FDR) upregulated genes are all annotated in AGPv3 as transposable elements, with 34 out of 40 on chromosome 4 and within a 2 Mb region containing the *ga1* locus but outside of the smallest 100 kb mapped region identified by Liu et al. (2014). To characterize these gene models further, homology was examined by BLAST. We examined the 34 genes from the *ga1* locus on chromosome 4 and all but one have pectinesterase/pectinesterase inhibitor 38, 17, or 46 as their top BLAST hit with the one remaining sequence having pectin methylesterase (PME) 63 as its top BLAST hit. The remaining six genes fall on other chromosomes and either have no significant BLAST hits, are related to kinesin (GRMZM2G327923) or nucleobase-ascorbate transporter 12 (GRMZM2G031728).

**Figure 3:**
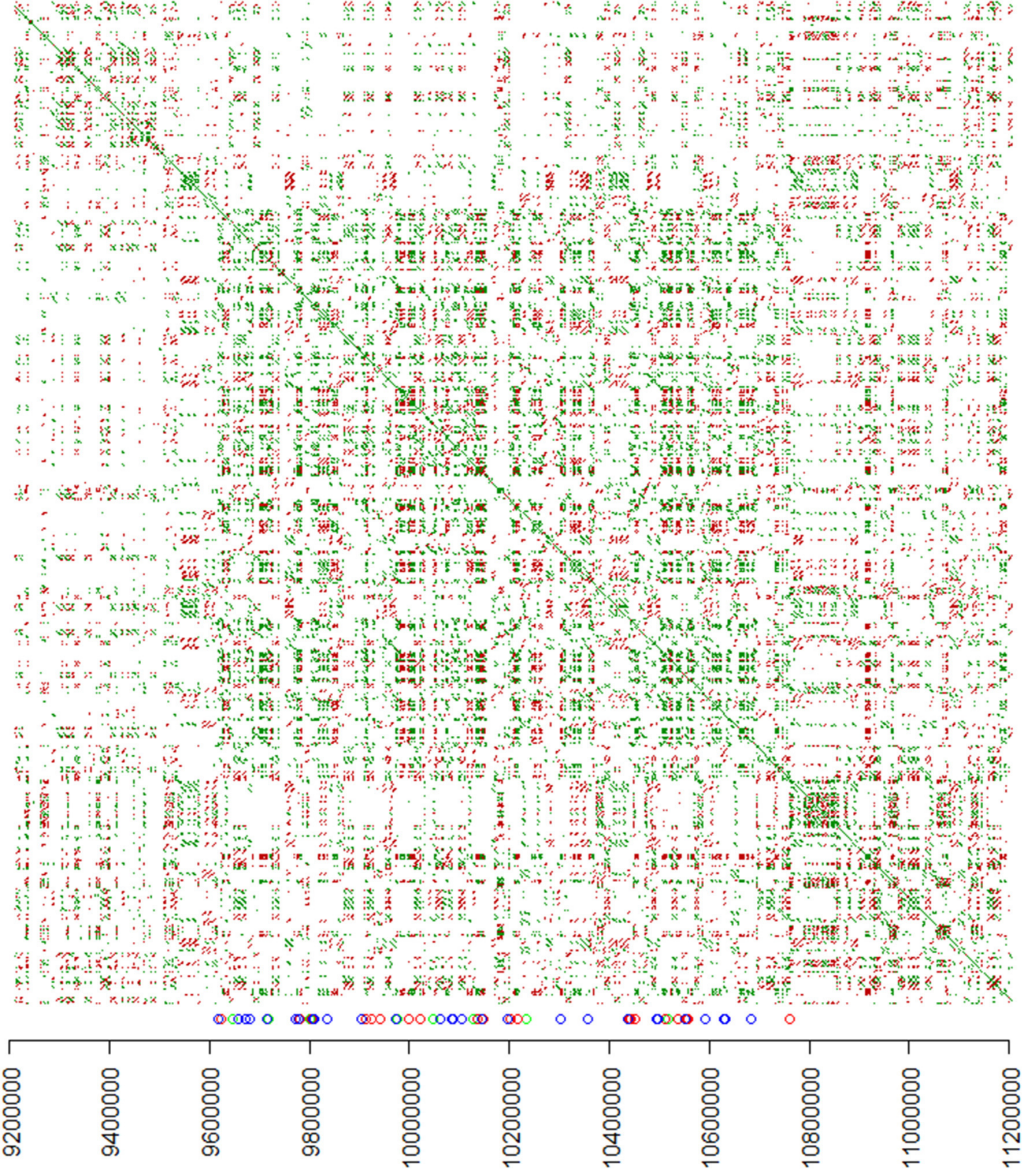
Structure of the PME repeat region in W22. A dot plot was constructed by plotting sequence matches with the indicated coordinates from the W22 chromosome 4 genomic sequence on both axes. Green dots indicate matching sequences on the forward strand and red dots indicate matching sequences on the reverse strand. On the X-axis, blue, green, and red circles indicate the positions of full-length, medium length, and short PME-like sequences, respectively.

**Figure 4:**
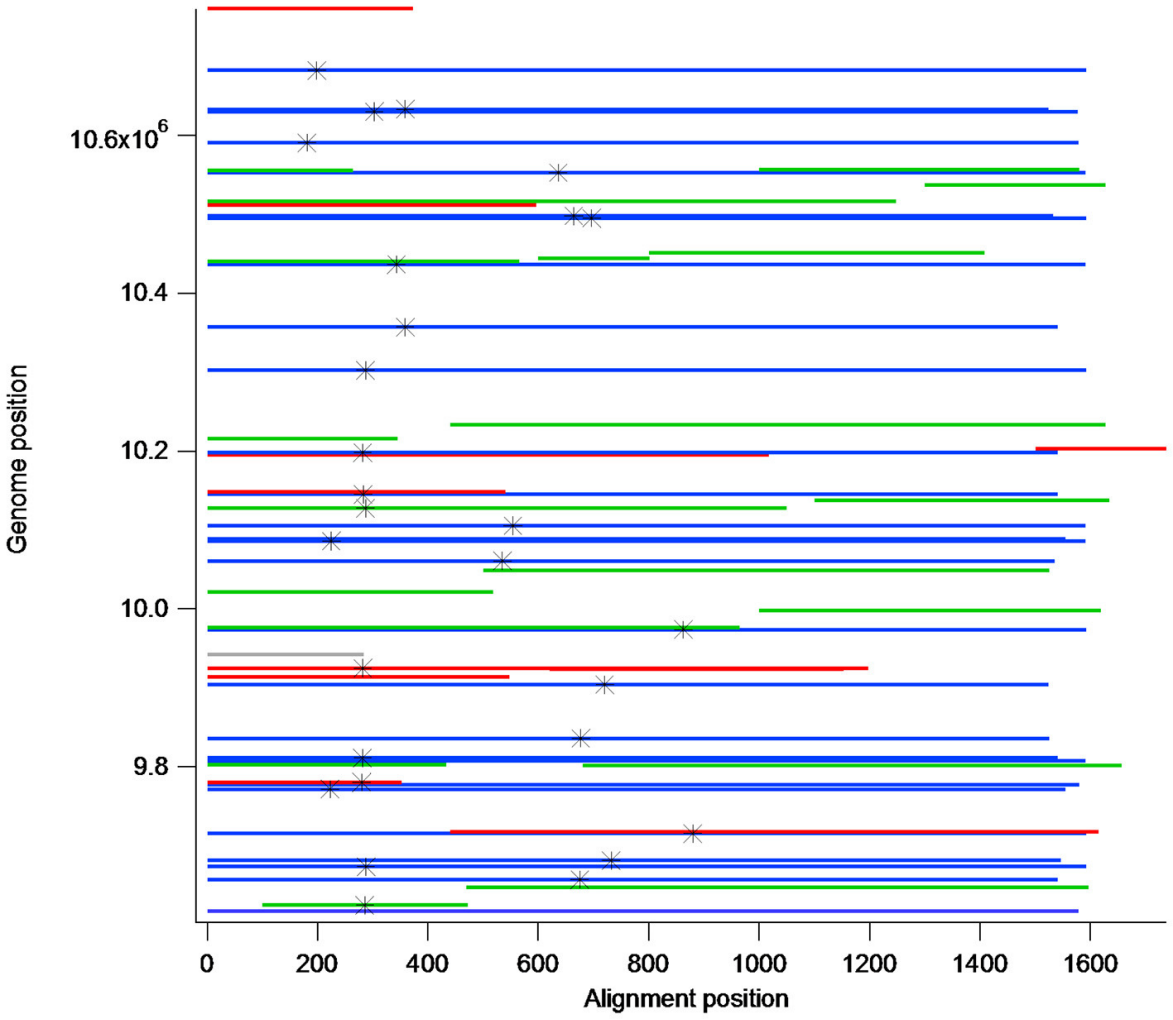
Alignment of 58 W22 PME-like sequences and their relative chromosome 4 genomic positions. Blue bars are full length PME-like sequences, green and red bars are middle and short length sequences, respectively. The positions of the first in-frame stop codon are indicated by an asterisk.

We compared the *de novo* assembled transcript to the B73 genomic sequence and the co-linear W22 sequence in the genetically mapped region of interest. In doing this, we found that this region contains a cluster of 58 PME-like sequences. In a 1.1 MB region of W22 there are 58 full length and partial PME genes (**Figure 4**; Table S2). All but three have pectinesterase/pectinesterase inhibitor 38/17 as their top BLAST hit. The remaining three have *Pme63* as their top hit, but appear to be partial genes. By aligning the 58 PME sequences from W22 and the assembled transcript, there does appear to be an intron in the W22 sequences. Although similar to the differentially expressed transcript, none of the B73 or W22 sequences matched it exactly. Using the ExPASy.org translate tool, we found that none of these genes have a full-length open reading frame, even accounting for an intron, while the *de novo* assembled transcript does. In several cases, a simple one base difference introduces a stop codon in the W22 sequence. All PME-like sequences in the gene cluster are oriented in the same direction. The 1.1 MB region containing the gene cluster has a complex organization of nested repeats (Figure 3), however the PME-like sequences are dispersed throughout the region with no obvious association with a larger repeat unit. Alignment of the PME-like sequences according to their genome position (Figure 4) shows that partial PME sequences are interspersed with complete sequences. In some cases the sequence organization suggests that partial sequences are derived from insertions into complete PME sequences because partial sequences adjacent to and on either side of some PME-like sequences are contiguous.

## Discussion

We report the discovery and characterization of a new maize pectinesterase gene and protein that is expressed in silks of *Ga1-s* but not *ga1* plants and maps to the *ga1* locus. We have named this gene *ZmPme3*. The genetic map position, *ZmPme3* allele sequences, RNA expression and protein expression are consistent with the hypothesis that ZmPME3 is involved in *Ga1* function. Additional work is needed to establish the role of ZmPME3 in the process of *Ga1* gametophytic incompatibility. Genetic disruption of *ZmPme3* and overexpression in transgenic plants, as well as heterologous expression and activity studies, could all be used to provide evidence of ZmPME3 function. Until these studies are complete, ZmPME3 should be considered a candidate gene for *Ga1* function. The observations that *Ga1* has different functions in different tissues suggest that the locus is functionally complex and presence of the *Ga1-m* allele suggests that it is genetically complex as well. Any molecular explanation of *Ga1* function will need to address these complexities.

PMEs have been shown to alter pollen tube growth in pollen tube cell walls in Arabidopsis (Jiang et al., 2005; Tian et al., 2006) and tobacco (Bosch et al., 2005; Bosch and Hepler, 2006). The likely mechanism for this activity is deesterification of homogalacturanan in the growing tip of pollen tubes. This alters the rigidity of the pollen tube cell wall, impairing its ability to grow directly toward the ovule. The growth behavior of pollen tubes with altered PME activity in these studies is consistent with the observations of incompatible pollen tube growth in *Ga1-s* silks presented here and elsewhere (Lu et al., 2014). An active version of *ZmPme3* is not found in either *Ga1-m* or *ga1* genotypes surveyed, which lack the *Ga1-s* female function. Genetic evidence suggests that the lack of the female function in *ga1* may be because *ga1* is a null allele of the *ga1* locus. Plants with the *Ga1-m* allele are able to pollinate *Ga1-s* but can also be pollinated by *ga1*. They therefore have the male function but lack the female function of the *ga1* locus. Our observation that *Ga1-m* contains a mutationally inactivated allele of *ZmPme3* would predict that inactivation of *ZmPme3* would result in the loss of the female function while the male function of *Ga1* is preserved. The male function is genetically separable from the female function in *Ga1-m*, suggesting that it is encoded by a separate but tightly linked gene from *ZmPme3*.

Interestingly, PMEIs are typically structurally similar to PMEs, but they contain an additional inhibitor domain on the N terminus of the protein (reviewed in Pelloux et al., 2007). This raises the possibility that the silk specific PME identified here is actually a PMEI and the transcript we identified is incomplete. Several observations suggest this is not the case. First, the intron/exon structure is more similar to a PME than a PMEI (Duan et al., 2016). Second, the gene encoding the differentially expressed silk PME contains predicted promoter elements including TATA and CAAT boxes. This suggests that the gene contains the transcriptional start site and we are not missing an exon containing a PMEI domain. Finally, the differentially expressed silk PME is predicted to contain a signal sequence. Such a sequence would target the PME to the extracellular space. This is consistent with ZmPME3 having a role in pollen exclusion which would require the PME to be present in the extracellular space to interact with pollen tubes. Since signal sequences are removed during translation, it is likely that the start codon identified here is correct.

## Conclusions

We have identified a pectin methylesterase gene designated as *ZmPme3* that is present in the silks of *Ga1-s* plants and maps to the *ga1* locus. This gene is lacking in *ga1* genotypes but present and mutationally inactive in *Ga1-m* lines which lack the female function of *Ga1-s*. We also identify a novel cluster of PME pseudogenes that map at the *ga1* locus in *ga1* genotypes. We propose a role for PME in the molecular mechanism of pollen exclusion that provides a framework for hypothesis testing and new potential targets for discovery in other maize gametophytic incompatibility systems.

## Accession numbers

The W22 genome sequence has been released under the Toronto Agreement, GenBank Bioproject PRJNA311133. RNA-Seq data is deposited in the NCBI Short Read Archive accession number SRP111127.

## Author contributions

AM analyzed and interpreted data, designed and performed the experiments, wrote and revised the manuscript. MM conceived of the work and generated the RNA-Seq data. RH acquired and interpreted data. MS conceived of the work, analyzed data, wrote and revised the manuscript. All authors contributed to the discussion and approved the final manuscript.

### Conflict of interest statement

The authors declare that the research was conducted in the absence of any commercial or financial relationships that could be construed as a potential conflict of interest.
