# Supplementary material for: A Pectin Methylesterase ZmPme3 Is Expressed in Gametophyte factor1-s (Ga1-s) Silks and Maps to that Locus in Maize (Zea mays L.)
Source: Front Plant Sci. 2017 Nov 7;8:1926. doi: 10.3389/fpls.2017.01926 (PMC5684833; doi:10.3389/fpls.2017.01926)
Supplement: Supplementary file 3 [file Table3.DOCX]

| **Table S3: High confidence differentially expressed genes mapped to B73_RefGen version 3.** | | | | | |
| --- | --- | --- | --- | --- | --- |
|  | **Gene ID** | **FDR** | **AGPv3 annotation** | **Top BLAST hit** | **Chromosome** |
| 1 | GRMZM2G057278 | 2.99E-115 | transposable element | PME38 | 4 |
| 2 | AC184840.4_FG002 | 7.68E-115 | transposable element | PME38 | 4 |
| 3 | GRMZM2G337471 | 1.14E-112 | transposable element | PME38 | 4 |
| 4 | AC191393.3_FG001 | 3.71E-112 | transposable element | PME38 | 4 |
| 5 | GRMZM2G123349 | 3.71E-112 | transposable element | PME17/38 | 4 |
| 6 | GRMZM2G431402 | 2.01E-111 | transposable element | PME38 | 4 |
| 7 | GRMZM2G124384 | 1.03E-109 | transposable element | PME38 | 4 |
| 8 | AC194254.3_FG004 | 2.63E-107 | transposable element | PME38 | 4 |
| 9 | GRMZM2G472020 | 5.52E-105 | transposable element | PME38 | 4 |
| 10 | GRMZM2G456385 | 4.16E-101 | transposable element | PME38 | 4 |
| 11 | GRMZM2G144454 | 5.72E-101 | transposable element | PME17/38 | 4 |
| 12 | GRMZM2G369162 | 1.55E-100 | transposable element | PME38 | 4 |
| 13 | AC205353.3_FG009 | 1.71E-100 | transposable element | PME17/38 | 4 |
| 14 | GRMZM2G088082 | 5.02E-100 | transposable element | PME38 | 4 |
| 15 | AC205353.3_FG008 | 2.39E-99 | transposable element | PME17/38 | 4 |
| 16 | AC191393.3_FG011 | 3.19E-99 | transposable element | PME38 | 4 |
| 17 | GRMZM2G369168 | 8.36E-99 | transposable element | PME38 | 4 |
| 18 | AC184840.4_FG011 | 5.11E-96 | transposable element | PME38 | 4 |
| 19 | AC184840.4_FG010 | 3.89E-95 | transposable element | PME38 | 4 |
| 20 | GRMZM2G068742 | 8.72E-95 | transposable element | PME38 | 4 |
| 21 | AC184840.4_FG004 | 1.06E-92 | transposable element | PME38 | 4 |
| 22 | AC191393.3_FG003 | 7.48E-90 | transposable element | PME38 | 4 |
| 23 | AC191393.3_FG012 | 1.16E-89 | transposable element | PME17/38 | 4 |
| 24 | AC194254.3_FG002 | 2.90E-85 | transposable element | PME38 | 4 |
| 25 | GRMZM2G404357 | 2.97E-85 | transposable element | PME38 | 4 |
| 26 | AC205353.3_FG004 | 1.58E-79 | transposable element | PME17/38 | 4 |
| 27 | GRMZM2G327923 | 1.48E-77 | low_confidence | kinesin | 3 |
| 28 | GRMZM2G702341 | 6.32E-77 | transposable element | PME38 | 4 |
| 29 | GRMZM2G463798 | 3.02E-75 | transposable element | PME17/38 | 4 |
| 30 | GRMZM2G046740 | 4.91E-69 | transposable element | n.s. | 4 |
| 31 | GRMZM2G380297 | 1.50E-68 | low_confidence | n.s. | 7 |
| 32 | GRMZM2G088100 | 2.45E-67 | transposable element | PME17/38 | 4 |
| 33 | AC203042.3_FG008 | 1.42E-65 | transposable element | PME63 | 4 |
| 34 | GRMZM2G041864 | 6.38E-64 | transposable element | n.s. | 2 |
| 35 | GRMZM2G314583 | 1.60E-63 | low_confidence | n.s. | 3 |
| 36 | AC205353.3_FG006 | 6.72E-63 | transposable element | PME38 | 4 |
| 37 | GRMZM2G031728 | 1.08E-60 | low_confidence | nucleobase-ascorbate transporter 12 | 6 |
| 38 | AC203042.3_FG010 | 3.60E-58 | transposable element | PME38 | 4 |
| 39 | GRMZM2G456387 | 2.08E-55 | transposable element | PME17/38 | 4 |
| 40 | GRMZM2G156046 | 1.45E-54 | transposable element | PME17/38 | 4 |
