# Supplementary material for: A Pectin Methylesterase ZmPme3 Is Expressed in Gametophyte factor1-s (Ga1-s) Silks and Maps to that Locus in Maize (Zea mays L.)
Source: Front Plant Sci. 2017 Nov 7;8:1926. doi: 10.3389/fpls.2017.01926 (PMC5684833; doi:10.3389/fpls.2017.01926)
Supplement: Supplementary file 5 [file Image1.pdf]

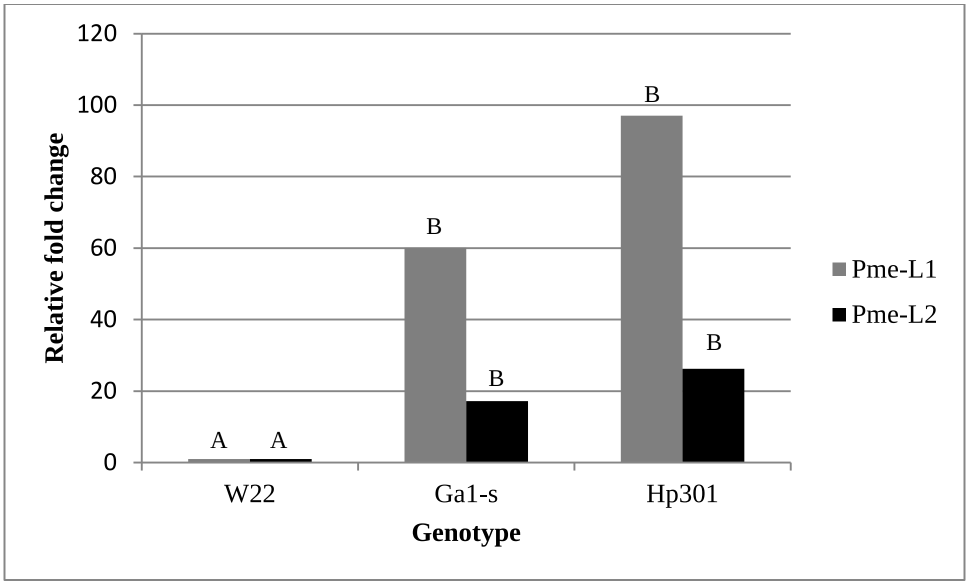

Figure S1: Relative RNA levels of *ZmPme3* in silks of *Ga1-s* (W22) and Hp301. Two primer pairs were used to confirm RNA expression of *ZmPme3* in *Ga1-s* genotypes and normalized to 18S RNA level. The $2^{-\Delta\Delta Ct}$ method was used to quantify RNA levels and expressed as relative fold change to W22 (*ga1*). While each differ in the relative fold change to W22 (*ga1*) levels, both show that *ZmPme3* is highly expressed in the silks of *Ga1-s* genotypes while the level detected in W22 was similar to NTC ΔCt levels. Significance groupings were determined by Student's t-test in JMP.
